# Reconciling the economic and biological fishery data gathered through the European Data Collection Framework: A new R-tool

**DOI:** 10.1371/journal.pone.0264334

**Published:** 2022-03-31

**Authors:** Isabella Bitetto, Loretta Malvarosa, Jörg Berkenhagen, Maria Teresa Spedicato, Evelina Sabatella, Ralf Döring

**Affiliations:** 1 COISPA Tecnologia & Ricerca s.c.r.l, Torre a Mare, Bari, Italy; 2 NISEA Fisheries and Aquaculture Economic Research, Salerno, Italy; 3 Thünen-Institute of Sea Fisheries, Bremerhaven, Germany; Hellenic Center for Marine Research, GREECE

## Abstract

Fishing fleets and targeted stocks are the basis for the design of multiannual management plans at European or Mediterranean levels. Management Strategy Evaluation and bioeconomic modeling need data at a specific level of resolution in terms of time, area and type of fishing activity for analyzing measures for management procedures using simulations. Within the Data Collection Framework, data are to be aggregated at different levels, e.g.: fleet segment and métier, the former linked to the predominant gear and the size of the vessel and the latter to the activity itself. Fishing costs are collected by fleet segment, effort and landings by fleet segment and métier. Bioeconomic modeling for management purposes requires data at the same resolution. The aim of this paper is to describe a methodology, implemented in SECFISH R package, to disaggregate variable cost data from the fleet segment to the métier level. The presented tool allows to determine the correlation between the variable costs of a vessel and its activities to estimate costs at the activity level (e.g. métiers). The tool is applied to selected Italian fleet segments characterized by a variety of métiers and high dynamicity.

## Introduction

In mixed fisheries, the interaction between fishing fleets and targeted fish stocks is the cornerstone for the design of multiannual management plans (MAP) for fisheries management at the European and Mediterranean levels [1–4]. On the other hand, the interaction between fleets and fisheries harvesting multiple stocks also faces several complexities [5, 6]. Management Strategy Evaluation (MSE) is a tool that scientists utilize in support of policies to simulate, through bioeconomic modeling, the functioning of a fisheries system and to explore the impact of several management measures for the achievement of pre-agreed objectives [4, 7]. MSE is also used to assess potentially negative consequences of existing or proposed regulations [8, 9].

Data availability is often a limiting factor for the design of a bioeconomic model, which adds complexity to the implementation [5]. Having the input information at the appropriate resolution level can make the difference in the selection of the model and in the level of accuracy of the assessment within the simulation framework.

In 2000, the European Union (EU) designed a specific framework to guarantee that each Member State carries out a regular collection and management of a wide range of fishery data needed for scientific advice in support of the Common Fisheries Policy (CFP). Within the Data Collection Framework (DCF, Commission Delegated Decision (EU) 2019/910), aimed at collecting information for fisheries assessment, bioeconomic modelling, scientific advice, different levels of aggregation for data provision are defined. In our context we focus on:

- fleet segment: a group of vessels with the same length class (LOA, length overall) and a predominant fishing gear during the year;
- métier: a characterisation of fishing operations targeting a similar (assemblage of) species, using similar gear, during the same period of the year and/or within the same area.

The economic information gathered by fleet segment includes operating costs that are linked to the activity of the vessel and fixed costs that incur independent of effort. The variable costs, together with other more stable components (e.g., fixed and maintenance costs), are included in the relevant economic indicators aimed at identifying the status of a fishery in terms of profitability (e.g., Break-Even Revenue (BER), Current Revenues/BER, Return on Fixed Tangible Assets (RoFTA), and Return on Investment (ROI)) [10] and are generally included in the assumption behind the bioeconomic models utilized for the design of management plans.

Most bioeconomic mixed fisheries models present in the literature also require data on the cost of fishing at métier level [5]. Specifically, the variable costs are generally estimated on the basis of effort, assuming a linear or quadratic function [11–13]. The importance of the métier in the bioeconomic models derives from the aggregation level for the biological data related to the demographic structures of the catches routinely used in stock assessment and, in turn, in bioeconomic modeling and MSE. However, no standardized and coded procedures to estimate the coefficients for deriving the variable costs associated to a specific effort are currently available in the literature.

A vessel can fish in different areas with different gears on different stocks over the year; hence, the overall sum of annual cost data only reflect an average across different fishing activities, not allowing to extrapolate the specificities of the different fishing activities that may relate not only to the impact on the marine resources (e.g., selectivity) [14] but also to differences in the operating costs’ structure.

Within the DCF, the fleet segment and the métier have been historically recognized as separate entities. Indeed, the so-called transversal data (i.e. fishing effort, weight and value of landings) are available by fleet segment and by métier, being strictly linked to the fishing activity, while the data on the fixed and variable costs are collected at the fleet segment level. While the fixed costs, e.g. insurance, are mainly related to vessel characteristics, the variable costs, i.e. mainly fuel and labor costs are more closely linked to the fishing activity.

As observed by the Planning Group for ECONomics [15] and STECF (Scientific, Technical and Economic Committee for Fisheries, the group of experts, appointed by the Commission to provide scientific advice on fisheries management) 16–02 [16], the resolution level of cost variables collected under DCF in several cases does not match end-users’ (e.g. STECF expert working groups) needs and is not sufficient for the main aim of the data collection, e.g., assessment of management measures through bioeconomic modeling. This is especially the case for variable costs, generally closely linked to the type of fishing activity exerted (e.g. métier). Nevertheless, in practice, options to increase the resolution of data are limited. Different fora (e.g. Planning Group for Economic Issues PGECON, STECF expert working groups) highlighted that it is practically impossible to get comprehensive cost data at higher resolution scales than “fleet segment per year”[17]. Data simply cannot be determined beyond a certain resolution level, e.g. the frequency of changing métiers might be higher than the frequency of fuelling. Thus, even a single fuel bill might represent a combination of métiers.

The uses of cost data for fisheries’ management strategies based on fishing effort regimes and spatially oriented multi-annual plans are manifold: a recent example is the application of BEMTOOL, IAM and SMART models for simulating the impact of different fishing effort regimes on Western Mediterranean demersal fisheries [4]. In similar applications, consistent fleet (group of vessels) and métier (type of activity) data are needed with a corresponding level of aggregation as a prerequisite for integrated bio‐economic management and advice. It is important to have data for bioeconomic models available at the required resolution [5] or to apply estimation techniques that allow data to disaggregate accordingly.

The main aim of this paper is to describe a cost-efficient methodology allowing to derive annual cost data at métier resolution through a linear disaggregation algorithm. The algorithm, based on the current available resolution of variable costs and transversal data in the DCF (i.e. at fleet segment level) is implemented in an R package and published on R Cran (https://CRAN.R-project.org/package=SECFISH). To illustrate the working mechanism of the method, the approach has been applied to selected Italian fleet segments, characterized by activities in different métiers.

## Materials and methods

The developed methodology consists of two phases: first, individual vessel data are used to derive the correlations between variable costs (fuel costs, labor costs) and transversal variables (effort, landings in value); second, the costs by fleet segment and the transversal variables by fleet segment and métier are used to assign the annual costs by fleet segment to specific métiers. Only the first phase requires access to individual vessel data, while the second phase was designed to be run by any end-user by access and use of publicly available information.

**Fig 1:**
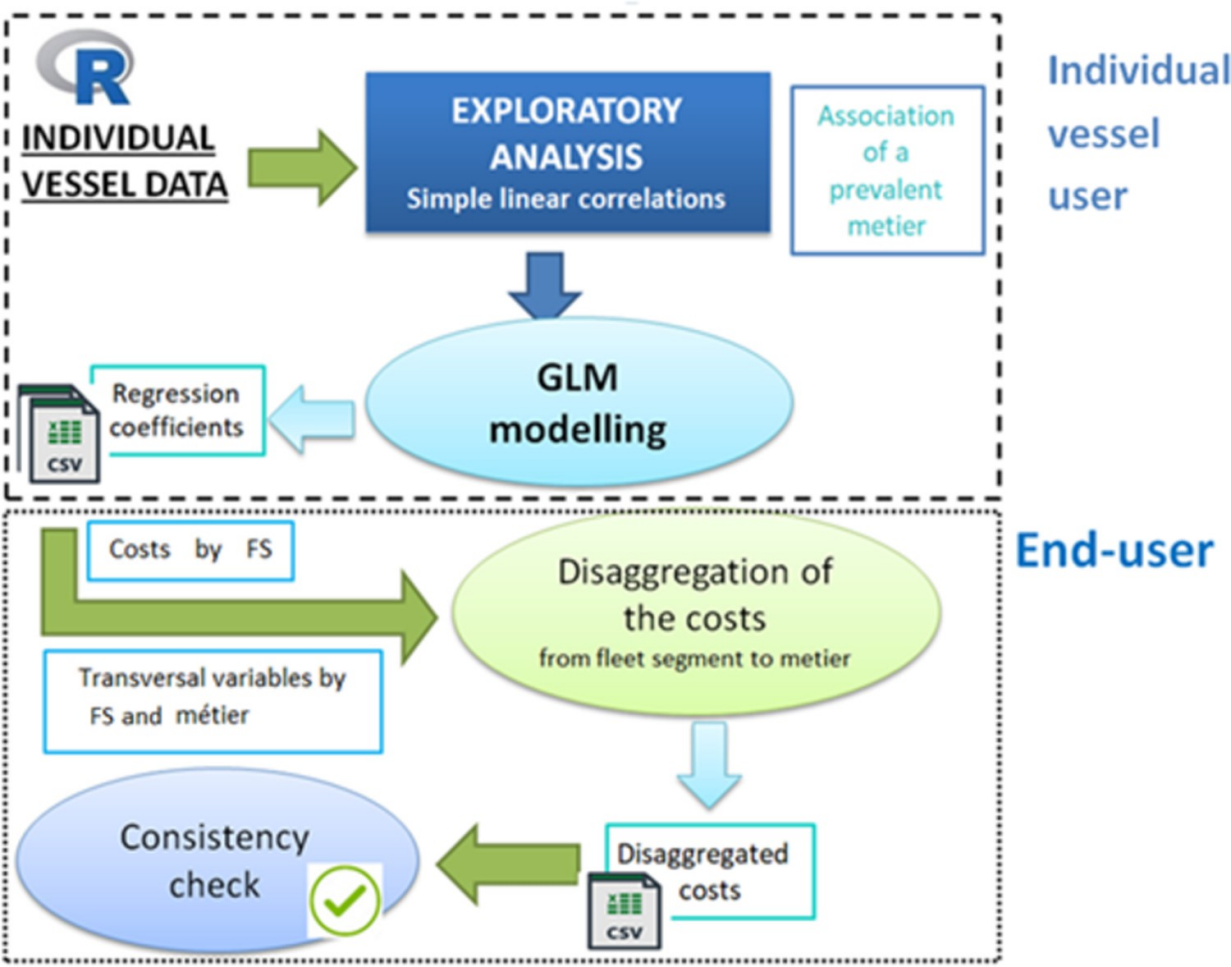
Workflow summarizing the functioning of the SECFISH package.

In Fig 1 the steps needed for the two phases are schematized. Firstly the individual vessel data are explored in order to identify for each vessel its prevalent métier; this association is used to group the individual data by prevalent métier. Then, simple linear correlations are applied to this new dataset to test the dependence of the variable costs on the transversal variable for each fleet segment within each métier. On the basis of the results of the simple linear models by métier, Generalized Linear Models (GLM, [18]) are applied for each fleet segment to test the significance of the métier categorical variable on the variable costs and to derive the coefficients for the disaggregation process.

In the second phase the coefficients derived in the first phase are, then, utilized to disaggregate the variable costs data, available at fleet segment resolution, at the métier level. Consistency checks are finally carried out between original data and disaggregated data.

Both costs by fleet segment and transversal data by métier are accessible through the EC data dissemination tool (https://stecf.jrc.ec.europa.eu/data-dissemination).

### Linear relationships between variable costs and transversal data

Firstly, the predominant métier is associated to each observation of the individual vessel data in order to obtain a new dataset, including the activity information and corresponding costs. According to phase 1 (Fig 1), within each fleet segment, Pearson’s correlation coefficient was estimated to derive first information about the strength of the link between the variable costs and transversal variables in the individual vessel dataset. As a further step, linear regressions (lm function of R package stats v3.6.2) were carried out, between the same variables, to statistically test the hypothesis of linear dependence of the costs on the transversal variables. A null intercept was assumed to simplify the disaggregation phase, but the linear model implemented in the R function can be easily generalized. The linear relationships considered for each fleet segment and métier are:

- Fuel costs vs. effort;
- Fuel consumption vs. effort;
- Labor costs vs. revenues (landings value) or vs. revenues minus total variable costs or vs. revenues minus fuel costs or vs. effort.

The effort can be expressed in terms of hours at sea or fishing days per KW.

This approach allows also to explore maintenance or other costs versus the effort relationships. However, we focused on “labor” and “fuel,” which are usually more closely linked to the transversal data (e.g., revenues and effort) and are the most relevant cost items for fishing vessels. The relationship between energy costs and effort have been already investigated during the Workshop on Allocation of Economic Data at disaggregated level as related to the DCF held in Hamburg in 2011 and the Workshops on European economic database and on disaggregation of economic data as related to the DCF held in Malta in 2012. [19–21] provided some elements concerning the link between labor costs and revenues. The analysis also comprises the fuel consumption to further elaborate the results obtained for fuel costs. Fuel consumption is the main driver for fuel costs, while the price must be considered an exogenous variable. Moreover, this variable, that is regularly collected within DCF, can be utilized to compare fleet segments regarding their respective fuel efficiency, in particular by spatially explicit bio-economic models [22].

For each combination “fleet segment-métier,” the significant dependence of the variable costs on the selected transversal variable (e.g., effort or value of landings) was tested. Evident difference among the slopes of the different métiers within a fleet segment is interpreted as a signal of possible different cost structure. Especially in this case, it could be relevant to statistically explore the métier effect on the costs for the purpose of disaggregation.

### Testing of the métier’s significance on variable cost structure

Proceeding within phase 1, we applied the generalized linear model (GLM) approach [16] with a Gaussian link function to investigate the significance of the métier, defined as a factor, on the cost structure within each fleet segment. The same relationships explored using the linear regressions were considered applying two different generalized linear models: the first aimed at testing separately the effect of métier and of transversal variables, and the second at exploring their combined effect (Eqs 1 and 2).

$$VC=\alpha *x_{1}+\beta *x_{2}+\varepsilon$$ Eq 1

$$VC=\alpha *x_{1}+\beta *x_{2}+\gamma *x_{1}x_{2}+\varepsilon$$ Eq 2

where VC (variable costs) is the response variable modeled by a linear function of explanatory variables *x*_1_ (métier, categorical), *x*_2_ (corresponding transversal variable, as numeric), and their combination *x*_1_*x*_2_, while the error is assumed to be distributed as a standard normal distribution. Furthermore, *α, β* and *γ* are the coefficients that are utilized to disaggregate the costs, if the métier is statistically significant.

In each fleet segment, the métiers representing a defined threshold (e.g. 80%) of the vessels’ activities observed have to be considered to avoid spurious relationships and convergence problems when fitting the GLM. The threshold can be set as an input in the corresponding function in the SECFISH R package.

### Selection of the model for disaggregation (phase 2)

**Fig 2:**
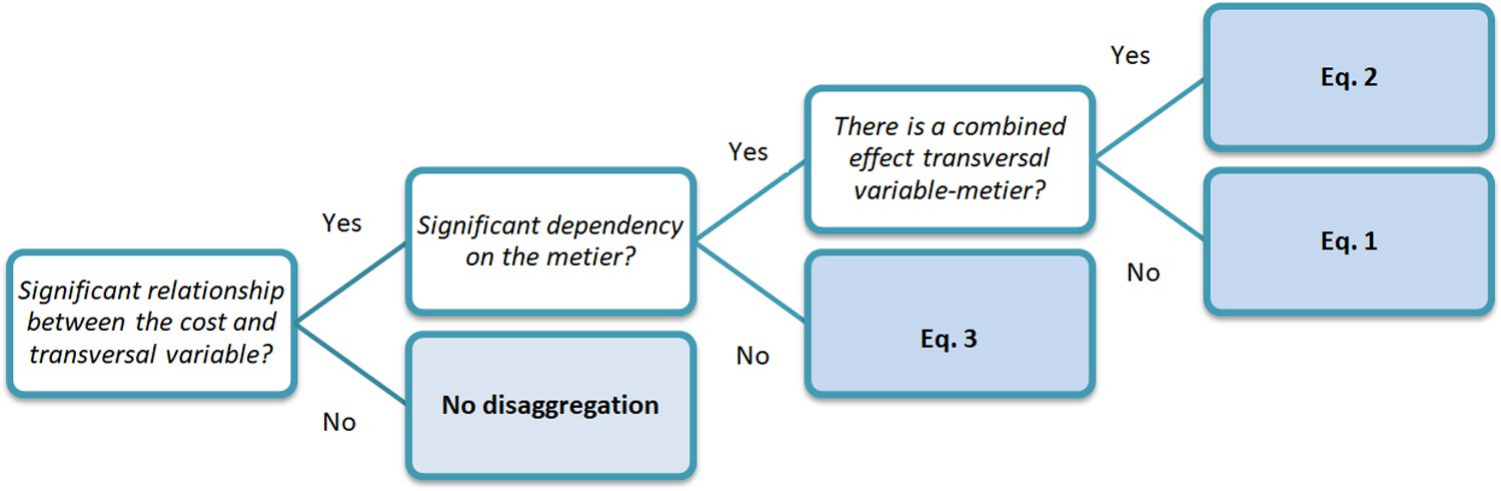
Decision support flow for the selection of the appropriate equation for the disaggregation of costs.

Fig 2 shows the decision support flow representing how to select the appropriate model for costs’ disaggregation, depending on the results of the phase 1. In the absence of a significant relationship between the costs and the transversal variables, the application of any disaggregation approach does not make sense. On the contrary, when a significant relationship is observed in the individual vessel data, it is then appropriate to inspect the GLM results in order to select the more appropriate model for disaggregating the costs.

According to the results of the GLM, three cases are then identified:

1. Dependency of variable costs on the transversal variables exclusively;
2. Dependency of variable costs on the métier and on the transversal variables separately;
3. Dependency of variable costs on the métier and on the transversal variables, separately and combined.

In case 1, the variable costs are assumed to vary linearly with the transversal variables equally for all the métiers:

$$VC=\alpha *x_{1}+\varepsilon$$ Eq 3

In cases 2 and 3, the disaggregation of the variable costs was carried out following the decision support flow in Fig 2 using Eq 1 or Eq 2, respectively.

When Eq 1 is selected, the disaggregated costs for a specific métier are derived summing up the coefficient corresponding to the métier (see Table 3, column Coefficient) to the coefficient related to the transversal variable multiplied by the transversal variable of the specific métier. Analogously if selecting Eq 2. If Eq 3 is selected, the costs by métier are derived as *α* multiplied by the transversal variable by métier, being *α* the same for all métiers. The variable costs by fleet segment were finally disaggregated according to the selected model, and a comparison between the official costs by fleet segment and the estimated costs by métier (summed up) was carried out as a consistency check.

### SECFISH R package

The above described methodology was implemented in SECFISH R package published on R Cran (https://cran.r-project.org/web/packages/SECFISH/index.html) through ad hoc functions that allow to apply all the steps reported in Fig 1. In order to cover the phase 1, the function EA allows to carry out an exploratory analysis on individual vessel data and to fit the linear regressions among the relevant type of costs and transversal variables, while the function GLM can be utilized to investigate the impact of métier on the cost structure of each fleet segment. Moreover, Disaggr and Cons_check functions were implemented to accomplish phase 2 and respectively the disaggregation of the cost data and the consistency check between the costs by fleet segment and by métier. In the package a dummy dataset is also provided, including a detailed description of the supported data format, both for individual vessel data and for the data to disaggregate.

The results of the analysis are provided through tables (e. g. GLM summary) and plots (e. g. linear regressions plots).

### Case study: Italian fleet

Individual vessel data on variable costs (fuel costs/consumption and labor costs) and fishing activity (e.g., duration of fishing activities by trip, métier used by fishing activity) were used to test the significance of the influence of the métier on the cost structure. 6,747 associated trips carried out by 519 Italian vessels, classified as belonging to the demersal trawlers (DTS) and hooks (HOK) fleet segments (Table 1) with different vessel lengths (VL), were monitored between the years 2014–2016, assigning the total number of fishing hours, landing volume and value to each trip. The data of the three years were pooled in the case study, thus the independence hypothesis of the observations could be violated. Indeed, the same vessel could be monitored in both years, although the corresponding data are interpreted as the two different observations. The métier and the fleet segments are indicated according to the DCF codification (https://datacollection.jrc.ec.europa.eu/wordef/fishing-activity-metier, https://datacollection.jrc.ec.europa.eu/wordef/fleet-segment-dcf). On one hand, fuel costs, labor costs and fuel consumption are linked to individual vessels; on the other hand, a prevalent métier is associated with each vessel according to the métier most frequently applied in terms of fishing activity, thus excluding those observations attributable to less relevant métiers. The variable costs associated with each observed vessel were then adjusted proportionally to the percentage of activity of the observations: if the prevalent métier represents the 75% for the total activity monitored for a vessel, all the type of variable costs associated to that métier for that vessel were estimated as the 75% of the variable costs collected for the vessel. By doing this, a new dataset of individual vessel data was generated, where each vessel is associated with a unique prevalent métier and its variable costs are approximately solely the ones due to that métier. Within each fleet segment a number of métier are, thus, present.

**Table 1:** Monitored Italian vessels by fleet segment and prevalent métier. In bold are the métiers representing 80% of the observations in the fleet segment (the 95% only for DTS_VL2440).

*Fleet segment*	*Fleet segment description*	*Metier* *	*Metier description*	*Number of observations* **
DTS_VL1218	Bottom trawlers with vessel length 12–18 m	GNS_DEF_> = 16_0_0	set gill nets	1
		GTR_DES_> = 16_0_0	trammel nets	1
		LLD_LPF_0_0_0	drifting longliners	3
		**OTB_DES_> = 40_0_0**	demersal trawlers	55
		OTB_DWS_> = 40_0_0	deep-water trawlers	26
		**OTB_MDD_> = 40_0_0**	mixed trawlers (deep and demersal)	53
		PTM_SPF_> = 20_0_0	Pelagic pair trawlers	4
		TBB_DES_0_0_0	beam trawlers	3
DTS_VL1824	Bottom trawlers with vessel length 18–24 m	LLD_LPF_0_0_0	drifting longliners	4
		**OTB_DES_> = 40_0_0**	demersal trawlers	51
		OTB_DWS_> = 40_0_0	deep-water trawlers	12
		**OTB_MDD_> = 40_0_0**	mixed trawlers (deep and demersal)	50
		OTM_MPD_> = 20_0_0	midwater otter trawlers	4
		PTM_SPF_> = 20_0_0	pelagic pair trawlers	3
		TBB_DES_0_0_0	beam trawlers	1
DTS_VL2440	Bottom trawlers with vessel length 24–40 m	**OTB_DES_> = 40_0_0**	demersal trawlers	39
		**OTB_DWS_> = 40_0_0**	deep-water trawlers	43
		**OTB_MDD_> = 40_0_0**	mixed trawlers (deep and demersal)	50
		PTM_SPF_> = 20_0_0	pelagic pair trawlers	1
HOK_VL1218	Vessels using hooks with vessel length 12–18 m	GTR_DES_> = 16_0_0	trammel nets	3
		**LLD_LPF_0_0_0**	drifting longliners	28
		LLS_DEF_0_0_0		8
		OTB_DES_> = 40_0_0	demersal trawlers	4
		OTB_MDD_> = 40_0_0	mixed trawlers (deep and demersal)	2
		PS_SPF_> = 14_0_0	purse seiners	1

* The numbers after the abbreviations represent the mesh size and other selective devices codification.

**Number of vessels with that prevalent métier observed in the dataset.

Following the second phase of the methodology, the DCF variable cost data of the selected Italian fleet segments were extracted from the EU Commission Fleet economic data repository (https://stecf.jrc.ec.europa.eu/dd/fleet), while the transversal data by fleet segment/metier have been extracted from the Fishery Dependent Information (FDI) data repository (https://stecf.jrc.ec.europa.eu/dd/fdi) for the years 2015–2016.

In the new dataset, the métiers representing 80% of the vessels’ activities observed in each fleet segment were considered, except for DTS VL2440 (95%), to avoid spurious relationships (bold métiers in Table 1).

## Results

### Linear relationships between variable costs and transversal data

The prevalent métiers identified in the DTS VL1218, DTS VL1824 and DTS VL2440 fleet segments are OTB_DES (bottom otter trawl for demersal species), OTB_DWS (bottom otter trawl for deep water species) and OTB_MDD (bottom otter trawl for mixed deep water and demersal species), while for HOK VL1218, it was LLD_LPF.

Table 2 shows the results of the linear relationships between variable costs and transversal data for each fleet segment and métier in terms of Pearson’s correlation coefficient, slope of linear regressions and significance. Only the métiers with a minimum of 30 observations were included in the analysis.

**Table 2:** Summary of results of the linear regressions by fleet segment and métier. In bold are the slopes with p-values <0.05. Only the métiers with a minimum of 30 observations were included in the analysis.

Fleet segment	Metier	Fuel costs		Fuel consumption		Labour Costs	
		Corr_coeff	slope	Corr_coeff	slope	Corr_coeff	Slope
**DTS_VL1218**	OTB_DES	0.4	**27.2**	0.5	**42.8**	0.9	**0.3**
	OTB_MDD	0.2	22.8	0.2	38.4	0.9	**0.3**
	ALL	0.4	**25.4**	0.5	**42.5**	0.9	**0.3**
**DTS_VL1824**	OTB_MDD	0.5	**32.2**	0.5	**55.9**	0.9	**0.3**
	OTB_DES	0.6	**42.3**	0.6	**76.3**	0.8	**0.2**
	ALL	0.5	**37.8**	0.6	**67.0**	0.8	**0.3**
**DTS_VL2440**	OTB_MDD	0.5	**40.4**	0.7	**77.2**	0.9	**0.3**
	OTB_DWS	0.4	**39.4**	0.5	**83.9**	0.9	**0.3**
	OTB_DES	0.2	41.2	0.4	**76.5**	0.9	**0.3**
	ALL	0.5	**40.1**	0.6	**80.4**	0.9	**0.3**
**HOK_VL1218**	ALL	0.2	12.5	0.3	22.5	1.0	**0.4**

**Fig 3:**
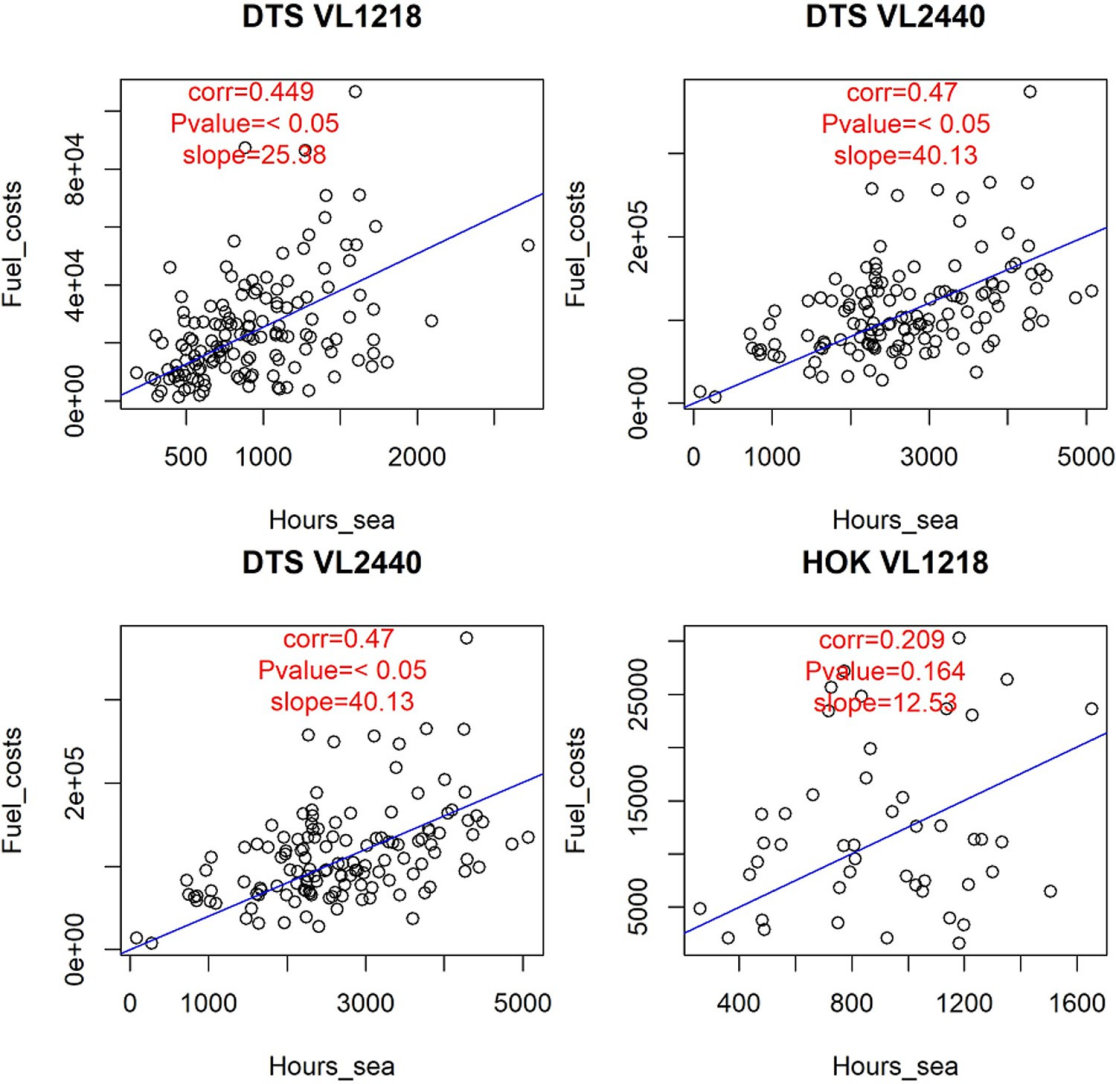
Linear correlations between fuel costs and hours at sea for the analyzed fleet segments.

**Fig 4:**
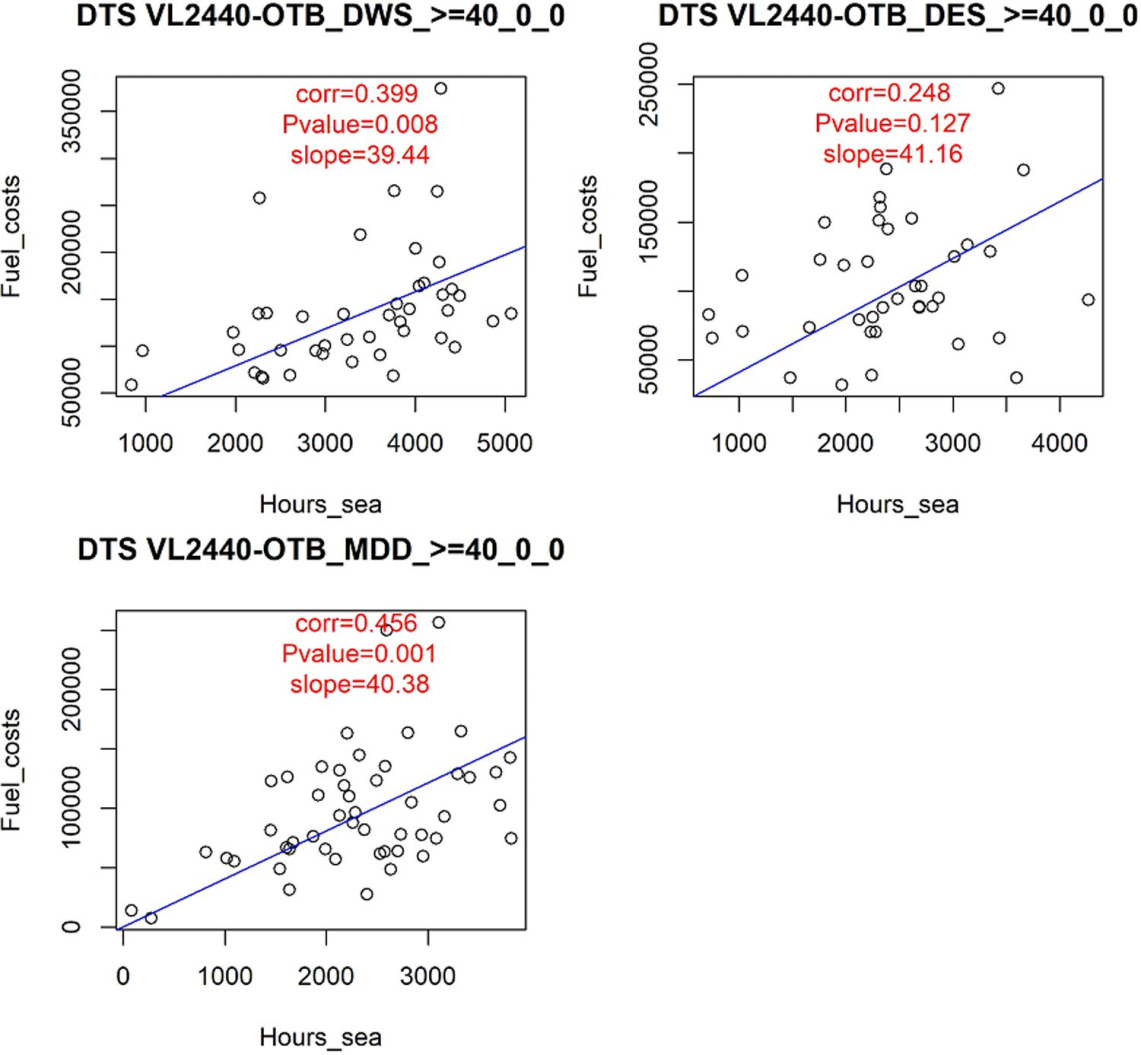
Linear correlations between fuel costs and hours at sea across the métier of DTS 2440.

**Fig 5:**
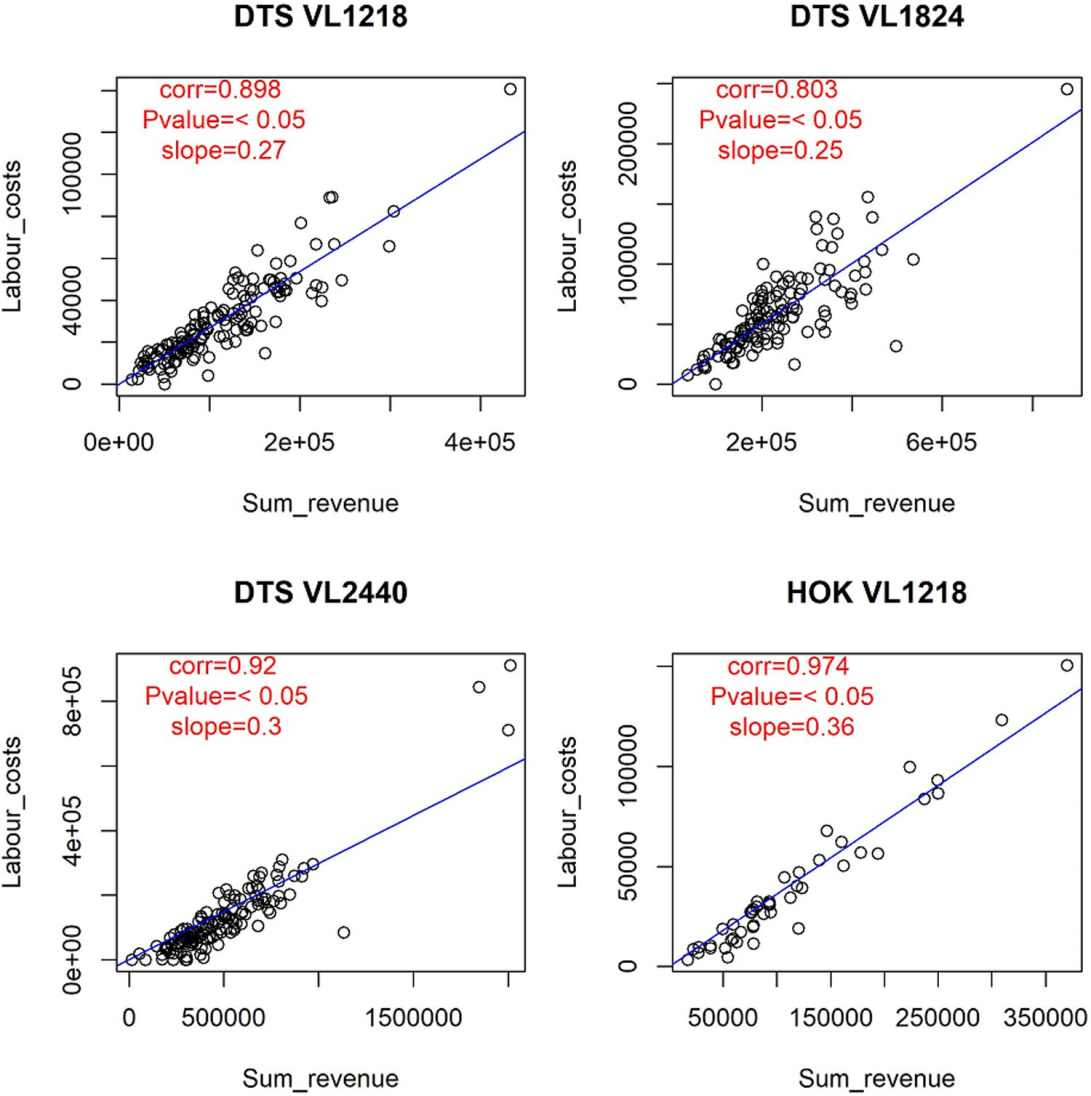
Linear correlations between labor costs and hours at sea for the analyzed fleet segments.

Figs 3–5 represent some of the graphical output of the SECFISH R package. In Fig 3 the results of the linear relationships between fuel costs and effort (hours at sea) are reported by fleet segment, not considering the métier. Concerning fuel costs, the results of the linear regressions highlight a significant (p-value <0.05) positive correlation with the hours at sea for all the fleet segments (Fig 3). Moreover, a significant dependency on almost all the métiers identified was observed for most fleet segments (Table 2). In Fig 4 the results of the linear regressions for the fleet segment DTS VL2440 by métier are reported. In particular, for fleet segment DTS VL2440, a strong correlation between the fuel costs and the hours at sea was found for each métier, with quantitatively different slopes (Fig 4). No significant influence was found for the métier OTB_MDD within DTS VL1218 and for OTB_DES within DTS VL2440. No significance was found within the fleet segment HOK VL1218 (Table 2).

Similarly, a significant dependency of fuel consumption on hours at sea was observed for all segments, except for DTS VL2440.

The labor costs are closely correlated with the revenues due to the crew share remuneration method applied by most Italian fisheries, [19–21] with a slope ranging between 0.3 and 0.4 (Table 2 and Fig 5).

### Testing the significance of the métier on variable cost structures

Table 3 reports the outcomes of the significance test of the métier through GLM. These results can be extracted by the GLM summaries that are stored by the SECFISH R package in dedicated directories by type of cost. Specifically, for the fuel costs, the fuel consumption and the labor costs the type of model that highlighted the significance of all the explanatory variables (among Eqs 1, 2 or 3), the coefficients α (for each métier) and β (for the effort), with their standard error, significance, explained variance of the model and Akaike Information Criterion (AIC) is indicated. Eq 2 was never selected in this case study. The output of the GLM shows that for all the trawlers’ fleet segments, the métier is a key factor significantly influencing, on one hand, fuel consumption and, as a consequence, fuel costs, with an explained deviance ranging from 67% to 85% and from 70% to 91%, respectively (Table 3). The strong correlation with the hours at sea was confirmed for all the examined fleet segments.

**Table 3:** Summary of results of the GLMs by fleet segment and métier. The results of the best performing option are reported for each cost type and fleet segment.

Type	Fleet segment	Expl. variable	Eq	Coefficient	Standard Error	Sign.	Expl. Dev.	AIC
***Fuel costs***	DTS_VL1218	OTB_DES	1	10165.8	4029.7	*	67	2421
		OTB_MDD		11687.3	4803	*		
		hours at sea		14.4	4.1	***		
	DTS_VL1824	OTB_MDD	1	14401.1	6942.5	***	82	2290
		OTB_DES		26058.9	7410.4	*		
		hours at sea		25.4	4.3	***		
	DTS_VL2440	OTB_MDD	1	49291.2	14455.4	***	85	3241
		OTB_DWS		57601.4	18407.9	**		
		OTB_DES		42976.9	13489.7	**		
		hours at sea		23.5	5	***		
	HOK_VL1218	hours at sea	3	12.5	1.2	***	70	961
***Fuel consumption***	DTS_VL1218	OTB_DES	1	13939.6	6143.4	*	70	2512
		OTB_MDD		18028.8	7322.4	*		
		hours at sea		25.3	6.3	***		
	DTS_VL1824	OTB_MDD	1	19310.694	11082.854	***	85.3	2290
		OTB_DES		47945.212	11829.872	.		
		hours at sea		46.494	6.841	***		
	DTS_VL2440	OTB_MDD	1	59430	20010	**	91	3342
		OTB_DWS		110700	27310	***		
		OTB_DES		67170	21450	**		
		hours at sea		7.4	7.19	***		
	HOK_VL1218	hours at sea	3	12.5	1.23	***	70	961
***Labour costs***	DTS_VL1218	Revenues	3	0.3	0.01	***	94	3072
	DTS_VL1824	Revenues	3	0.3	0.01	***	91	2844
	DTS_VL2440	OTB_MDD	1	-48730	8976	***	88	3225
		OTB_DWS		-57820	12340	***		
		OTB_DES		-59370	10110	***		
		Revenues		0.3	0.01	***		
	HOK_VL1218	Revenues	3	0.4	0.01	***	97	966

Signif. codes: 0 ’***’; 0.001

’**’; 0.01

’*’; 0.05 ’.’; 0.1 ’ ’

In all the fleet segments, labor costs were not influenced by the métier, but only by the revenues, except for DTS VL2440, for which a significant impact of the métier on the costs was found. For DTS VL1218, DTS VL1824 and HOK VL1218, the revenues appear to be the main explanatory variable, with an average explained deviance of 94%.

### Selection of the model for disaggregation

Following the workflow in Fig 2, the best performing model was selected with respect to the results of linear correlations and GLM for each fleet segment and for each type of variable cost (Table 3).

For fuel costs and fuel consumption, Eq 1 was found suitable for all the DTS fleet segments, with the métier and hours at sea being the explanatory variables. For HOK VL1218, hours at sea were the unique explanatory variable for fuel cost, as only one prevalent métier could be observed for this segment.

Only for the DTS VL2440 fleet segment was the métier found to also have an influence on labor costs, while for the other fleet segments, these costs were driven uniquely by the revenues.

### Disaggregation of the variable costs and consistency checks

**Fig 6:**
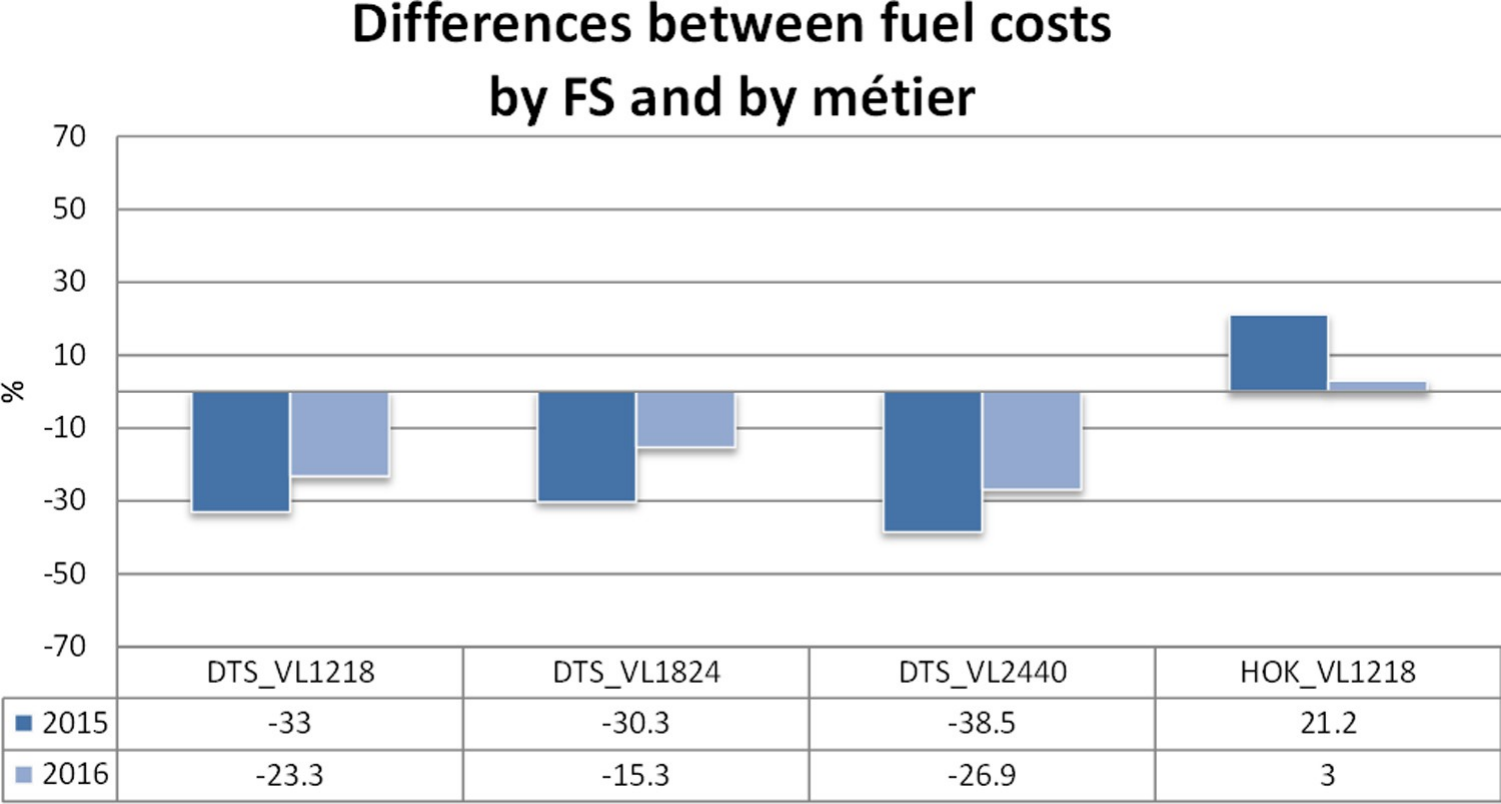
Differences between the fuel costs by fleet segment and by métier.

**Fig 7:**
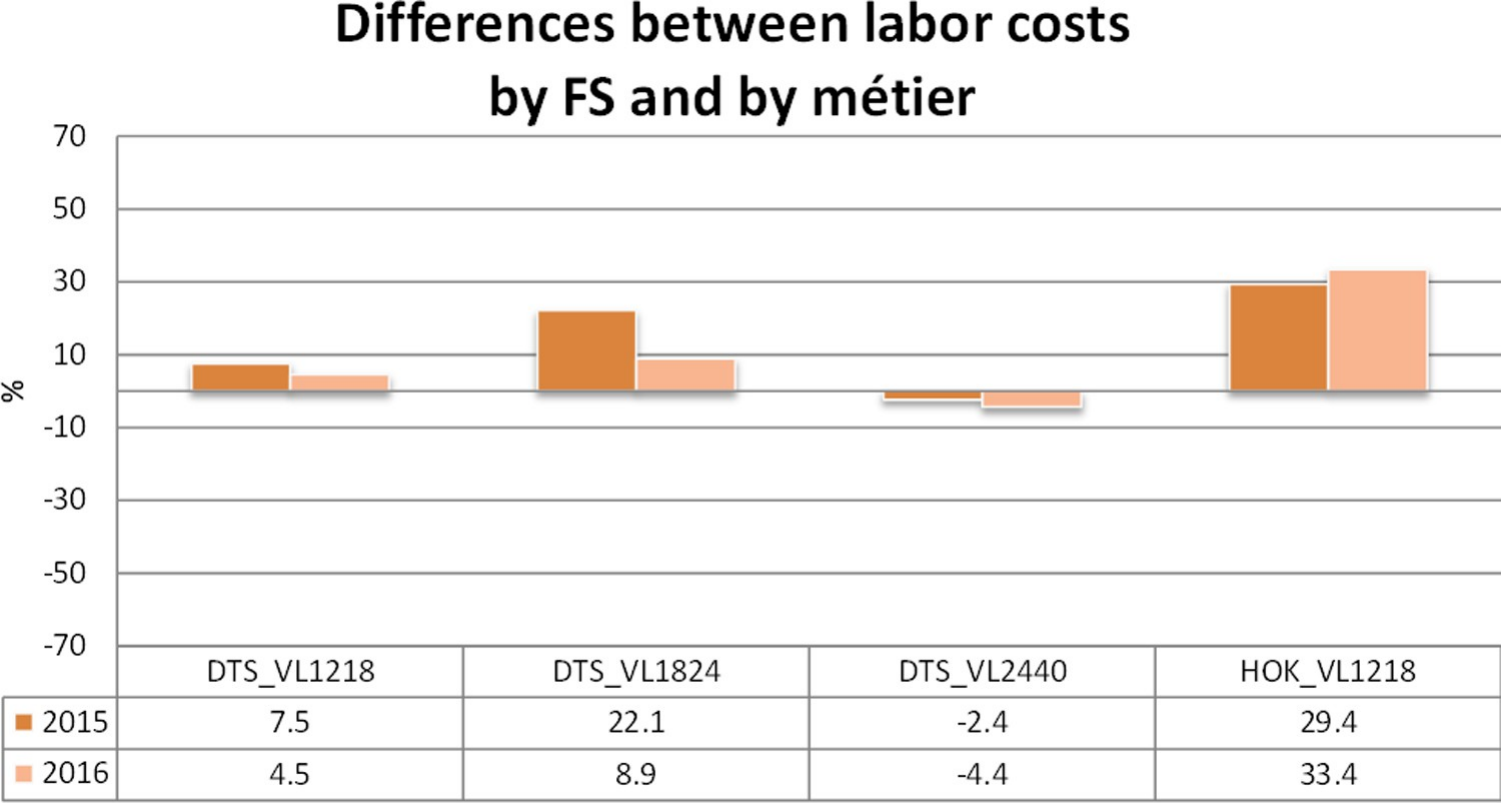
Differences between the labor costs by fleet segment and by métier.

The variable costs were disaggregated according to the relationships (and corresponding coefficients) reported in Table 3 for the years 2015 and 2016. When Eq 1 is selected, the coefficients in Table 3 were used, while when Eq 3 was selected, the slopes in Table 2 were considered. Table 4, representing the outcome of the disaggregation function in the SECFISH R package, reports the comparison between the original costs by fleet segments and the sum of the disaggregated costs. In Figs 6 and 7 the same differences are represented both graphically and through percentages of the original costs by fleet segment. The comparison between the costs by fleet segment and the sum of the estimated disaggregated costs by métier (Figs 6 and 7 and Table 4) showed that the disaggregated costs exhibit an overall relative difference of around 19% in absolute values. This difference can be explained, considering that for the estimation of correlations for each fleet segment, only the individual vessel data related to the métier representing 80% of cumulative fishing activity were included in the analysis to avoid convergence problems. These problems occurred in the development phase because of the inclusion of very few observations related to not prevalent and poorly represented métiers within the fleet segment, lastly determining the impossibility of properly testing the métiers’ influence on the fleet segment costs.

**Table 4:** Results of the consistency checks between variable costs by fleet segment and variable costs disaggregated by métier.

*year*	*Fleet_segment*	*variable_name*	*Sum_costs_by_metier*	*Costs_by_fleet_segment*
**2015**	DTS_VL1218	fuel_costs	32 724 856.52	48 857 605.00
**2016**	DTS_VL1218	fuel_costs	32 939 217.59	42 962 358.00
**2015**	DTS_VL1218	labour_costs	47 387 361.97	44 073 843.00
**2016**	DTS_VL1218	labour_costs	46 853 510.47	44 856 310.00
**2015**	DTS_VL1824	fuel_costs	41 411 282.73	59 385 350.00
**2016**	DTS_VL1824	fuel_costs	43 208 396.67	51 021 250.00
**2015**	DTS_VL1824	labour_costs	45 782 917.51	37 494 387.00
**2016**	DTS_VL1824	labour_costs	47 788 448.40	43 892 592.00
**2015**	DTS_VL2440	fuel_costs	16 699 956.82	27 161 577.00
**2016**	DTS_VL2440	fuel_costs	18 717 106.16	25 610 939.10
**2015**	DTS_VL2440	labour_costs	27 341 877.83	28 002 571.00
**2016**	DTS_VL2440	labour_costs	26 310 929.14	27 535 993.57
**2015**	HOK_VL1218	fuel_costs	3 000 661.00	2 475 812.00
**2016**	HOK_VL1218	fuel_costs	2 519 331.00	2 445 413.00
**2015**	HOK_VL1218	labour_costs	5 355 868.54	4 140 049.00
**2016**	HOK_VL1218	labour_costs	5 361 530.78	4 018 552.00

For DTS VL1218 and DTS VL2440, larger differences between cost by fleet segment and the sum of estimated costs by métier were obtained for the fuel costs, while the sum of the disaggregated labor costs seems to be globally more in line with the figures by fleet segment. For DTS VL1824, the 2015 data are less consistent than the 2016 data for both labor and fuel costs.

## Discussion

Several bioeconomic models require variable costs as input at the resolution of metiers or comparable levels. However, these cost data are usually only available at higher aggregation, namely at fleet segment level as defined under EU data collection legislation, thus hampering the application of the models. As a consequence, the structure of variable costs has to be assumed homogeneous for all métiers throughout a fleet segment when applying these models.

In order to overcome this issue, individual vessel data on activities by métier need to be explored. This investigation can also be useful to obtain a more accurate characterization of the fleet, not only based on the technical characteristics of the vessels but also on the habits of the fishermen in terms of visited fishing areas and métiers.

Within bioeconomic models, the variable costs are generally assumed to be proportional to the effort. However, no standardized procedure to estimate the coefficients, describing this linear dependency, is currently published.

The methodology described here allows both to carry out this investigation on individual vessel data in order to facilitate the identification of the main metiers exerted by the fleet segments and to explore whether and how the structure of their variable costs depends on factors other than vessel length and main gear. This deeper insight in cost structures should improve the quality of bioeconomic projections.

In order to apply the methodology presented here, annual cost and fishing activity data by métier are needed for individual vessels. In addition to aggregated cost data, information needed includes the type of fishing activity at trip level and the cost data for the sampled vessels. Hence, access to individual data is crucial in order to apply the method. Because this type of data, although possibly anonymized, are expected to potentially contain sensitive information, they could be obtained through a formal request to Member States, specifying the purpose of their use. This drawback could be overcome, utilizing specific assumptions on the crew share (for labor costs) and on the fuel cost/consumption per unit of effort; these assumptions can be based on literature or expert knowledge, rather than on individual vessel data.

The method is not limited to fleet segments and métiers. It can be adapted to any level of fishing activity and vessel grouping for which cost data are available at higher level, as long as activity data are accessible at the required resolution.

The focus of the present work was on energy (fuel) and labor costs; these are indeed the major components of the operating costs, the former being the major ones for some active gears, like demersal trawls, and strictly linked to the fishing time, while the latter depend mostly on the revenues. Nonetheless, the methodology is applicable for any type of variable cost.

An application to the Italian trawlers and longliners fleet segments, which are characterized by a marked dynamism and mobility in terms of fishing activity [23], is now presented to show the suitability of the developed methodology.

It should be noticed that, on one hand, the methodology facilitates the disaggregation of the variable costs in the presence of dependency both on the métier and on the transversal variables; on the other hand, it helps identifying the relationship between costs and transversal variables in the case of a unique prevalent métier or non-dependence on the métier. Concerning these two macro-groups, the results showed a different structure in the energy costs analysis, while a similar dependency of the labor costs on the revenues was detected for both. This is consistent with the crew share system widely used in most Italian fisheries.

Regarding the trawlers fleet segments, the relevance of the métier on the energy costs seems consistent with the characteristics of the explored métiers; indeed, for smaller and medium sized trawlers (vessel length between 12 and 24 meters), the métier targeting demersal resources and the one targeting demersal and deep-water resources represent quite different types of activity. While the demersal fishing activity is generally carried out on the continental shelf, producing lower energy costs, the mixed one tends to be distributed offshore with longer steaming times, causing higher fuel consumption and thus energy costs. The difference between the métiers is more explicit for large trawlers (length>24 meters), where even the deep-water métier, characterized by a more specific deep-water species target, is well represented.

For these fleet segments, the selected models explain a satisfactory amount of the variability in the data and allowed to disaggregate the fuel costs by metier, with a percentage of difference from the aggregated official values of around 19%; this is still consistent with the choice to consider only the metiers representing 80% of activity in the individual vessel data to avoid convergence problems.

For the fleet segment composed by vessels mainly using passive gears such as longlines (coded under HOK VL1218), it was not possible to properly test the significance of the métier on fuel and labor costs because, according to the individual vessel data, this fleet segment exerts only one prevalent métier in the individual vessel data.

The results for labour costs generally highlight the strong dependency on revenues, as expected for the crew share remuneration system applied in Italy. Indeed, only for large trawlers (length>24 meters) the métier was found to have an impact on the crew share. This is due to the higher variability in fishing activity within this particular fleet segment (three prevalent métiers). Indeed, for example, a trip of a trawler targeting deep-water species (OTB_DWS) generally can last more than one day, as it is carried out in more distant fishing grounds; this is different from the trip of trawler just targeting demersal species (OTB_DES), which is performed closer to the port of origin. It is not surprising that the salary needs to fit the duration of the fishing trip along with revenues.

Moreover, this methodology allows not only to highlight the dependency of the variable costs on the type of métier utilized, but also on the fishing grounds most commonly visited. Indeed, the application of the model to the Belgian fleet (not presented here but preliminary developed during the ICES WKTRADE2 [24] showed a different structure of the variable costs according to the area exploited, especially in terms of fuel costs. The integration of the fuel cost structure, linked to the fishing area, might allow for designing spatial management measures through bioeconomic modelling. This would thus permit the evaluation of different management strategies in terms of environmental footprints under various climate change scenarios [25].

The identification of the fishing grounds preferred by a fleet segment and the availability of spatially disaggregated variable costs would open the possibility, integrating the allocation of the sensitive areas of relevant stocks (e.g., nursery areas), of determining the biological and socio-economic effects of specific spatial closures (e.g., Fishery Restricted Areas) [4].

The possibility of obtaining variable costs at a higher resolution would, in combination with a raster of effort or revenues data by fishing ground or grid cell, allow to also predict the fleet adaptation/effort displacement starting from historical data [23]. This outcome could be used within spatial bioeconomic models (e. g. [26] (SimFish in the North Sea) and [27, 28] (DISPLACE in the North Sea or the Adriatic Sea)) to explore the reallocation of fishing efforts between different gears or métiers as a key aspect in the strategy adopted by fishers in reaction to management measures.

From this perspective, the development of this methodology could represent a step forward to the possibility of properly testing spatial fishing restrictions in fishery by accounting, in a meaningful way, for fleet adaptation/effort displacement and drivers in individual fisher decision-making in a meaningful way [23].

The focus of this paper is on active gears (trawls), for which the fishing effort can be quite precisely expressed in terms of hours at sea and kW. More exploration is necessary for analyses on passive gears other than hooks, such as gillnet and trammel net, for which the effort needs to be defined according to more specific variables, e.g., for number of trips.

A necessary disclaimer for the proper use of the method presented here is related to the completeness of the input data: in order to allow a reliable analysis, the individual vessel dataset used for deriving the regression coefficients needs to be large enough and properly represent the distribution of the effort by métier within the whole fleet segment. The more individual vessel data represent the fleet segments for which a disaggregation is desired (in terms of proportion of fleet segments and métier), the more, the estimated costs by métier are expected to be consistent with the data available at the aggregated level.

Another limit of the analysis is addressed by pooling individual vessel data from different years. This has the advantage of increasing the sample size. However, especially if more than three years are grouped, the independence hypothesis of the observations could be violated. To account for this dependency structure, it may be advisable to include a vessel random effect or a time series model (e.g., AR1), when modeling the GLM on individual vessel data, for counting a “year effect.” Moreover, other distributions for the GLM could be explored in the future for a positive continuous response variable (e.g., a Gamma distribution with a logarithmic link function) in order to relax assumptions of normality in the residuals. Based on these considerations, the developed methodology can be extended to accommodate the modeling of the costs for vessels applying passive gears and to account for random effects.

## Conclusion

The variable costs of a fishery represent a component of the expenses that can be controlled by vessels’ owners to increase the cash flow and that cannot be generally disregarded by any modeling approach of a fishery.

In order to properly utilize the available data collected under the European Data Collection Framework within bioeconomic models for the evaluation of fishery management measures, it is crucial to reconcile the economic and biological fishery data resolution.

The methodology described here has been implemented in the SECFISH R package and allows to derive annual cost data to the métier level and/or eventually to the fishing area level through a linear disaggregation algorithm based on variable costs and transversal data. It is a cost-efficient technique as it is intended to produce more disaggregated cost data without changing the resolution scales currently implemented in the DCF, hence not increasing the financial budget supporting the data collection at MS level.
